# Comparative analysis of jujube and sour jujube gave insight into their difference in genetic diversity and suitable habitat

**DOI:** 10.3389/fgene.2024.1322285

**Published:** 2024-02-06

**Authors:** Lingzhi Shao, Ping Qiao, Jingyi Wang, Yanfang Peng, Yiheng Wang, Wenpan Dong, Jie Li

**Affiliations:** ^1^ School of Biology and Food Science, Hebei Normal University for Nationalities, Chengde, China; ^2^ Dexing Research and Training Center of Chinese Medical Sciences, China Academy of Chinese Medical Sciences, Dexing, China; ^3^ State Key Laboratory for Quality Ensurance and Sustainable Use of Dao-di Herbs, National Resource Center for Chinese Materia Medica, China Academy of Chinese Medical Sciences, Beijing, China; ^4^ Laboratory of Systematic Evolution and Biogeography of Woody Plants, School of Ecology and Nature Conservation, Beijing Forestry University, Beijing, China

**Keywords:** jujube, sour jujube, plastome, genetic diversity, rapid identification, divergence time, suitable habitat

## Abstract

Jujube (*Ziziphus jujuba* var. *jujuba* Mill.) and sour jujube (*Z. jujuba* var. *spinosa* (Bunge) Hu ex H.F.Chow.) are economically, nutritionally, and ecologically significant members of the Rhamnaceae family. Despite their importance, insufficient research on their genetics and habitats has impeded effective conservation and utilization. To address this knowledge gap, we conducted plastome sequencing, integrated distribution data from China, and assessed genetic diversity and suitable habitat. The plastomes of both species exhibited high conservation and low genetic diversity. A new-found 23 bp species-specific Indel in the *petL-petG* enabled us to develop a rapid Indel-based identification marker for species discrimination. Phylogenetic analysis and dating illuminated their genetic relationship, showing speciation occurred 6.9 million years ago, in a period of dramatic global temperature fluctuations. Substantial variations in suitable climatic conditions were observed, with the mean temperature of the coldest quarter as the primary factor influencing distributions (−3.16°C–12.73°C for jujube and −5.79°C to 4.11°C for sour jujube, suitability exceeding 0.6). Consequently, distinct conservation strategies are warranted due to differences in suitable habitats, with jujube having a broader distribution and sour jujube concentrated in Northern China. In conclusion, disparate habitats and climatic factors necessitate tailored conservation approaches. Comparing genetic diversity and developing rapid species-specific primers will further enhance the sustainable utilization of these valuable species.

## 1 Introduction

**FIGURE 1:**
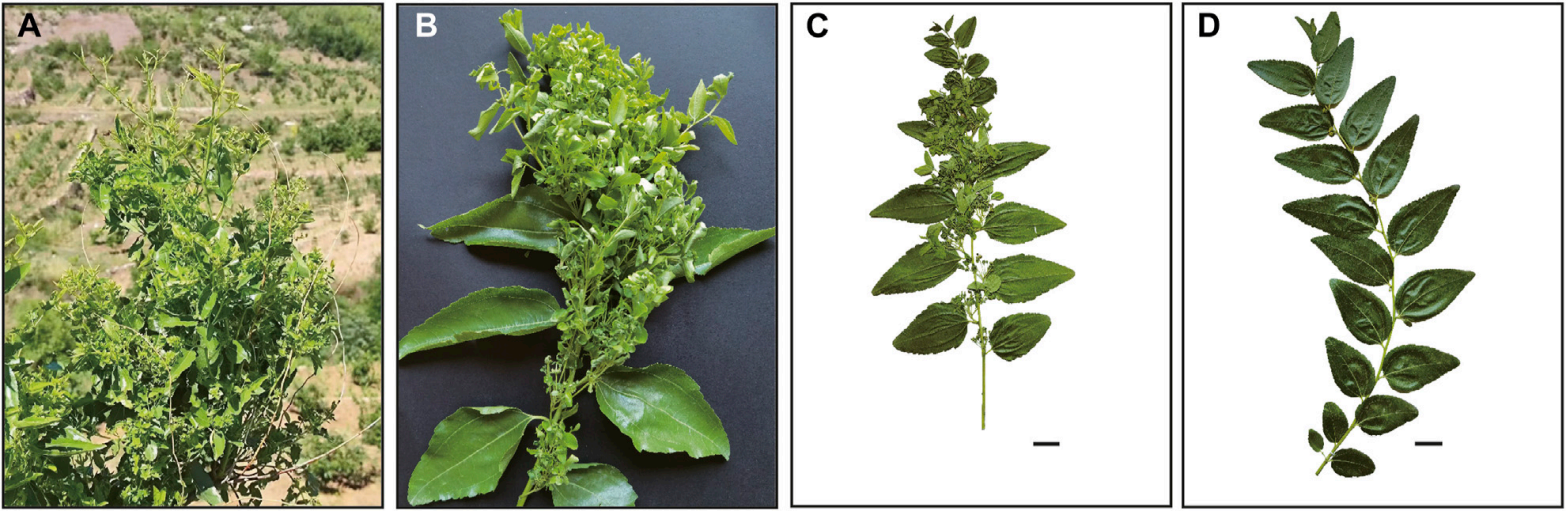
Morphological branches characters of jujube affected by JWB disease **(A–C)** and normal branch **(D)**. The scale bar represents 2 cm.

According to Our World in Data 2019 (https://ourworldindata.org/grapher/anxiety-disorders-prevalence), about 3.8% of the global population is living in anxiety in 2019, and 3.2% of Chinese have symptoms of anxiety disorder. The kernel of the sour jujube (*Z. jujuba* var. *spinosa* (Bunge) Hu ex H. F. Chow.) is one of the most frequently used traditional Chinese medicines (TCM) for releasing anxiety in prescriptions. The sour jujube has a sibling variety, the cultivated jujube (*Ziziphus jujuba* var. *jujuba*), which is a well-known fruit eaten fresh or dried and has medicinal and other values (Liu et al., 2013). It has an annual yield of approximately 8.52 million tons in China in 2017 (Guo et al., 2020). Unfortunately, the crop often suffers from jujube witches’-broom (JWB) disease (Figure 1), which significantly reduces yields. Such a disease has rarely been seen on sour jujube, which is widespread in the temperate regions in China, especially prevalent in cold, dry and barren habitats from the Loess Plateau to the Taihang Mountains (Du et al., 2022). It is often used as a rootstock for jujube for its tough vitality and high resistance to JWB disease. Besides its anti-anxiety effect, the sour jujube is believed to have sedative and tranquilizing, anti-aging, and anti-depression, antitumor, and myocardial protective functions (Sun et al., 2011).

Based on Flora of China (Yilin Chen and Schirarend 2007), there are three varieties and a form under the jujube species *Z. jujuba* Miller. Besides the two varieties mentioned above, the third variety is var. *inermis* (Bunge) Rehder without spines and the form is f. *tortuosa* C. Y. Cheng and M. J. Liu with tortuous branches. Both var. *inermis* and f. *tortuosa* are actually mutants under cultivation (cultivars), while var. *jujuba* has larger drupe of sweet taste and acute stones and var. *spinosa* is nearly completely wild with small drupe of sour taste and obtuse stones. This indicates that var. *jujuba* and var. *spinosa* are mainly distinguished by the identification of drupes. However, due to frequent phenotypic variations, field identification and distinction between these two species, particularly at the seedling stage, becomes more challenging (Ya qiang 2016).

The differences between the two major varieties under *Z*. *jujuba*, var. *jujuba* and var. *spinosa*, would be due to either long evolutionary histories or intra-varietal polymorphisms. Unfortunately, there is still a lack of a solid phylogeny of jujube genus and the genetic relationships of *Z*. *jujuba* to other species remain to be revealed. Luckily, determination of plastid genome has become routine recently and the complete plastid genome (plastome) sequences have been widely employed in phylogenetic reconstructions for their small genome size, mostly single copy, uniparental transmission, no genetic recombination, moderate evolution rate and satisfactory resolution, and cost-effectiveness (Dong et al., 2021a; Dong et al., 2021b; Wang et al., 2022b; Dong et al., 2022).

*Ziziphus jujuba* var. *spinosa* was once treated as distinct species as named *Ziziphus acidojujuba* C. Y. Cheng et M. J. Liu (Liu and Cheng, 1994). Its specific status remains to be phylogenetically tested based on plastid genome data. The alternative hypothesis that var. *jujuba* originated from var. *spinosa* by human selection implies that there is considerable genetic diversity within var. *spinosa*. The genetic diversity of both var. *jujuba* and var. *spinosa* has been well evaluated separately using microsatellite markers (Zhang et al., 2015; Nabavi et al., 2019) or SNP (Du et al., 2022). Sadly, no comprehensive comparative studies of both varieties have been carried out and the pedigrees of var. *jujuba* remain to be traced.

Considering that jujube fruits have become raw materials of many industries, suitable habitat zone prediction is essential for long term production of both geo-authentic raw medicine and fruits of high quality. MaxEnt is a software for modeling species niches and potential distributions using maximum entropy methods (Phillips and Dudík, 2008). It has been widely used for simulating suitable distribution habitat and building connections between climate and distribution (Kong et al., 2021; Zhang et al., 2021). It also associates genetic diversity to climatic factors which shape the levels and patterns of genetic structure of populations (Ortego et al., 2015). Understanding the correlations of genetic diversity with climatic factors or geographical patterns are the prerequisite in conservation of genetic diversity.

Phylogeny, genetic diversity and ecology are three indispensable aspects in evolutionary biology. Phylogeny provides more sense to biology in the light of ancestor-descendant relationship (Lee, 1995). Genetic diversity is the basis of ongoing evolution (Pauls et al., 2013). Ecological factors are external driving force in evolution and shape the geographical patterns of genetic diversity (Fine, 2015). In this study, we first built a solid phylogenetic relationships among related species of *Z. jujuba* based on plastome data and estimated the divergence times of lineages. Then we assessed the genetic diversity of jujube and sour jujube, seeking the possibility to distinguish them using DNA sequences or DNA barcodes. Thirdly, we modeled the potential habitats of both jujube and sour jujube based on specimen information and found out their optimal habitats. We aim to determine 1) the phylogenetic relationship of *Z. jujuba* with closely related species, 2) the progenitor-descendent relationship of jujube and sour jujube and possibility to discriminate them, and 3) the major factors shaping their geographical patterns of genetic diversity for the sustainable use and conservation of jujube genetic resources.

## 2 Materials and methods

### 2.1 Plant material and DNA extraction

Fifteen jujube accessions, comprising five accessions of var. *jujuba* and ten accessions of var. *spinosa*, were newly collected for plastome sequencing (Supplementary Table S1). Additional nine plastomes (seven var. *jujuba* and two var. *spinosa*) were retrieved from Genbank (Supplementary Table S1). Four closely related species in genus *Ziziphus* were downloaded from Genbank and taken as outgroups for further phylogenetic reconstruction (KY628304, MN017132, OP480228, and MZ475300).

A set of sixteen samples (eight var. *jujuba* and eight var. *spinosa*) were collected from field for species specific barcode verification. All the voucher specimens were identified by Jie Li and are now deposited at Hebei Normal University for Nationalities. Total genomic DNA was extracted using a modified cetyl trimethyl ammonium bromide (mCTAB) method (Li et al., 2013), and subsequently purified using the Wizard DNA Cleanup System (Promega, Madison, WI, United States). DNA quality assessments were performed using a 1% (w/v) agarose gel, and stored at −20°C.

### 2.2 Sequencing, assembly, and annotation

Following fragmentation into 350 bp by ultrasound, we prepared a paired-end library using the NEBNext UltraTM DNA library prep kit (New England Biolabs, Ipswich, MA, United States). PE150 sequencing was conducted on the Illumina HiSeq XTen platform at Novogene Co., Ltd. (Beijing, China). Raw data were subjected to filtering using Trimmomatic 0.39 software to generate clean data for subsequent assembly (Bolger et al., 2014). The Getorganelle v1.7.5 software was employed for *de novo* assembly of clean data with the following settings: F embplant_pt, -R 15, and -k 85,105 (Jin et al., 2020). As a further quality check, all reads were mapped to the assembled plastome using Geneious 8.1 (Kearse et al., 2012). The online platform CPGAVAS2 was conducted for gene annotation using a self-contained database (2,544 plastomes) as reference (Shi et al., 2019). The maps of the plastomes were drawn by the online program Chloroplot (https://irscope.shinyapps.io/Chloroplot/).

### 2.3 SNPs, indels, and hotspot identification

Plastomes were aligned using MAFFT online (https://mafft.cbrc.jp/alignment/server/) and manually checked using MEGA v7. Summary statistics regarding genome size, GC content, the sizes of the four regions, and the gene count for all 24 plastomes were generated in Geneious 8.1. Polymorphic sites, haplotypes, haplotype diversity (Hd), indels, and nucleotide diversity (Pi) for each species were calculated using DnaSP v5.10 (Librado and Rozas, 2009). Nucleotide diversity and indels were calculated within 500-bp sliding windows, with indels quantified with the “Multiallelic” gap option. A circos plot was generated using the “OmicStudio” tools online platform (https://www.omicstudio.cn/tool/) based on Pi and indel count data to visualize plastome hotspot regions. Species-specific variations, encompassing single nucleotide polymorphisms (SNPs) and indels, were manually enumerated in MEGA v7. Visualization of the IR/SC boundary maps for the two species was performed using IRscope (Amiryousefi et al., 2018). Species-specific primer synthesis and Sanger sequencing were both performed at the Sangon Biotech (Shanghai, China). The quality and size of PCR products was evaluated using a 2% (w/v) agarose gel. Sanger sequencing results were presented by the R package “ggmsa” (Zhou et al., 2022).

### 2.4 Phylogeny and divergent time estimation

A total of 28 plastomes (24 jujube accessions and four outgroups) were used for phylogenetic reconstruction. The maximum likelihood (ML) and Bayesian inference (BI) methods were carried out for phylogeny reconstruction. The program ModelFinder was used to select the best-fit model using BIC criterion. The maximum likelihood (ML) tree was generated using IQ-TREE (Nguyen et al., 2015), implementing the TVM + F + I model within PhyloSuite (Zhang et al., 2020), with branches having bootstrap values below 50 collapsed using TreeCollapseCL (Drinkwater and Charleston, 2014). The BI tree was constructed using MrBayes 3.2.6 with a GTR + I + F model for 500,000 generations (Ronquist et al., 2012). The initial 25% of sampled data were discarded as a burn-in. Trees were displayed in FigTree v1.3.1 (http://tree.bio.ed.ac.uk/software/figtree/).

For divergence time estimation, the BEAST v2.6.6 platform (Bouckaert et al., 2019) was employed, utilizing a relaxed log-normal clock model with a GTR substitution model and a speciation Yule Process tree prior. Given the absence of a reliable fossil point within the genus *Ziziphus*, a secondary calibration point (Z. mauritiana, 15.4 mya) was utilized for dating (Guo et al., 2021). A Markov Chain Monte Carlo (MCMC) chains for were performed (500,000,000 generations, sampled every 10,000). The effective sample size (ESS) was assessed to ensure all parameters exceeded 200. Following a burn-in of 25%, a maximum clade credibility (MCC) tree with 95% highest posterior density intervals was computed at each node using TreeAnnotator 2.1.3 (BEAST packages). The final trees were displayed and modified in FigTree v1.3.1 and Adobe Illustrator CS6.

### 2.5 Distribution occurrences and habitat modeling

The distribution occurrences of var. *jujuba* and var. *spinosa* in China were downloaded from the Global Biodiversity Information Facility (GBIF). For distribution records quality assurance, we generated the respective distribution maps for each species in ArcGIS 10.8, manual check and removed the points inconsistent with the flora description. Subsequently, spatial rarefaction was applied with a 10 km resolution using the SDM toolbox v2.5 to minimize sampling deviation during simulation, resulting in 311 records for var. *jujuba* and 185 records for var. *spinosa* (Brown et al., 2017).

Nineteen climatic variables, representing current climate conditions with a 2.5′resolution, were obtained from WorldClim (https://www.worldclim.org/). To prevent overfitting of the models, a Pearson correlation analysis was performed using R package “ggpair” among the 19 climatic variables for each species, and pairs with |r| > 0.7 were removed (Emerson et al., 2013). This process yielded five climate variables for var. *jujuba* and six for var. *spinosa*. The cross-validation method was applied by randomly selecting 75% of sites for model training in ten replicate runs, with the remaining 25% used for validation (Zhang et al., 2021). Model robustness was assessed using the Area Under the Curve (AUC) value, which ranges from 0 to 1. Evaluation criteria for AUC values were as follows: poor (0.6–0.7), fair (0.7–0.8), good (0.8–0.9), and excellent (0.9–1) (Zhao et al., 2021; Wang et al., 2022a). The calculation result of the MaxEnt modeling implies potential suitable rate for distribution, and with a value also ranged from 0 to 1. To interpret these values, we adopted an equal interval approach and categorized potential suitability into five levels: no suitability (0–0.2), low suitability (0.2–0.4), medium suitability (0.4–0.6), high suitability (0.6–0.8), and ultrahigh suitability (0.8–1) (Wang et al., 2023). Geographic areas falling into these different suitability levels were computed using ArcGIS 10.8 and further adjusted in Adobe Illustrator CS6.

## 3 Results

### 3.1 Plastome features and variation

Fifteen *Ziziphus* plastomes were generated using a genome skimming approach and have been released in GenBank with corresponding accession numbers and detailed information, as provided in Supplementary Table S1. All 24 plastids, consisting of the fifteen newly sequenced and nine downloaded from GenBank, exhibited a typical quadripartite structure comprising two Inverted Repeat (IR) regions (26,436–26,515 bp) separated by the Large Single Copy (LSC) region (88,897–89,428 bp) and the Small Single Copy (SSC) region (19,356–19,369 bp). The genome size ranged from 161,211 bp (KX266830) to 161,819 bp (OR438246). The average GC content, at 36.8%, displayed no significant variation among the 24 accessions of var. *jujuba* and two var. *spinosa*. Both species’ plastomes contained 112 unique genes, encompassing 78 protein-coding genes, 30 transfer RNA genes, and four ribosomal RNA genes. The lengths of the IR regions in both species were conserved, with only minor differences observed and no significant expansion or contraction of IR boundaries (Supplementary Figure S1).

For a precise understanding of genetic diversity, we estimated them at specific level (24 plastomes) and variety level (12 plastomes each) with detailed information provided in Supplementary Table S2. Excluding sites with gaps and missing data, the total aligned length for genetic diversity estimation at species level was 160,558 bp (Supplementary Table S2). There were 16 haplotypes with a haplotype diversity (Hd) of 0.913, 140 indels and 258 variable sites. The average nucleotide diversity (Pi) was calculated as 0.00032. In var*. jujuba*, the total length was 160,966 bp and there were six haplotypes (Hd = 0.682), 96 indels and 202 variable sites (Pi = 0.00021). In var*. spinosa*, the total length was 160,858 bp and there were 10 haplotypes (Hd = 0.955), 39 indels and 64 variable sites (Pi = 0.00007) (Supplementary Table S2).

### 3.2 Hotspot identification and species-specific barcodes

**FIGURE 2:**
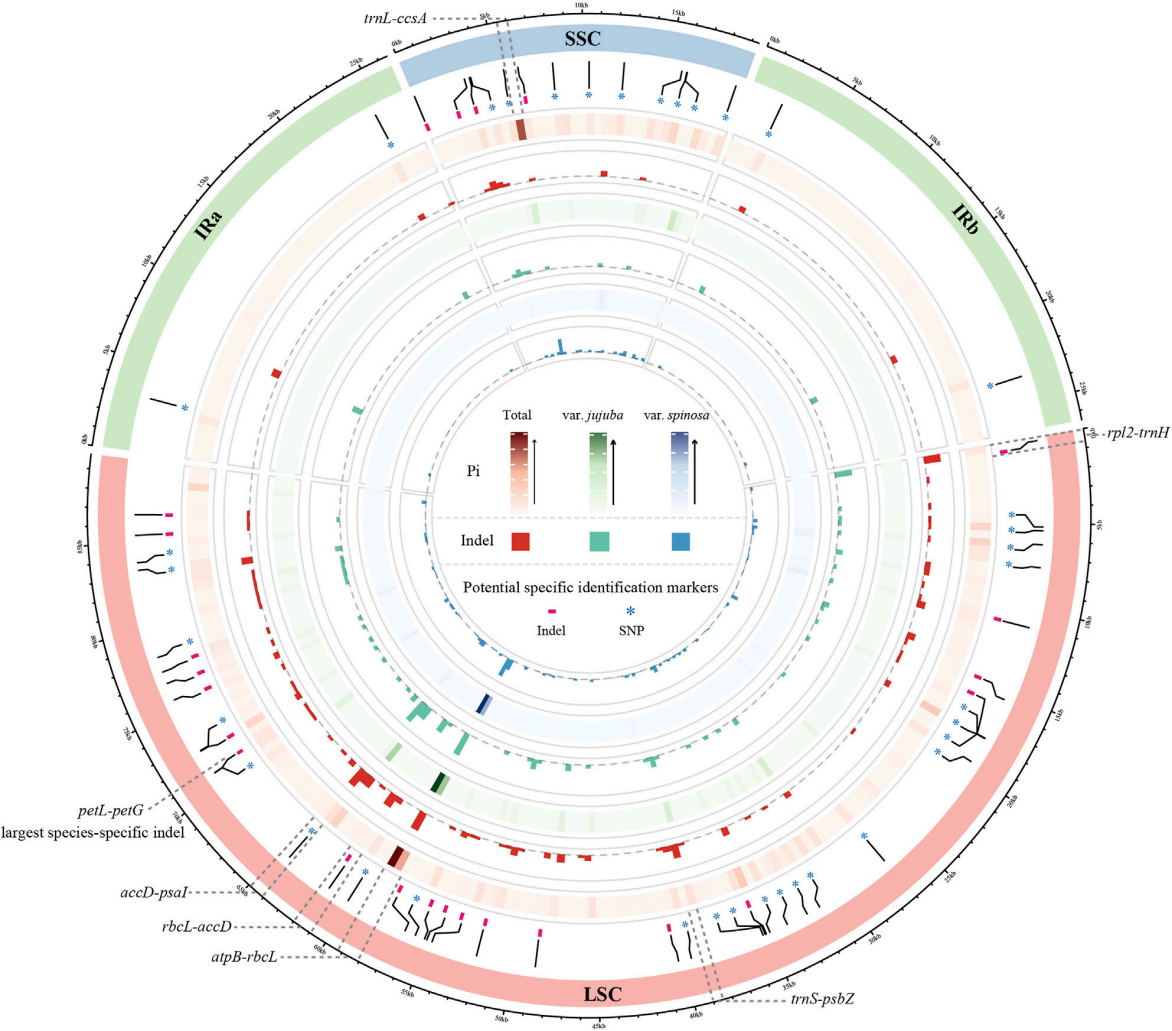
Comparison circos plot of plastomes showing the indel and nucleotide diversity. Circles from the outer to inner area show the following: structure of plastomes indicated by different colors; species-specific SNP and Indel labeled by ‘*’ and ‘-’; nucleotide diversity shown by a heatmap and indel count shown by a histogram of three dataset, in order, total group, only for jujube, and only for sour jujube. Window size of each grid is 500 bp.

To visualize the patterns of Pi and indels across the three datasets, we constructed a Circos map utilizing sliding window analysis, with each grid window size set at 500 bp (Figure 2). The Pi of a single window reached up to 0.00933 in total group, 0.009 in var. *jujuba*, and 0.00867 in var. *spinosa*. Indels within a single window exhibited variability, ranging from zero to seven in both the total group and var. *jujuba*, and from zero to six in var. *spinosa*. Based on the criteria of indel greater than five or Pi exceeding than 0.007, we identified a total of six hotspot regions, which have been clearly labeled in the figure. Notably, the region encompassing *rbcL-accD* exhibited the highest indel count and the greatest Pi. In general, the regions exhibiting variation in indels closely mirrored those with varying Pi, and these identified hotspot regions, as indicated in the figure, were predominantly located within the Large Single Copy (LSC) and Small Single Copy (SSC) regions. Remarkably, all of these hotspot regions were positioned within spacer regions.

**FIGURE 3:**
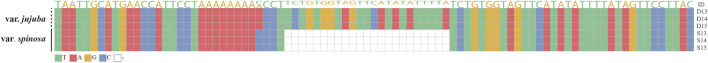
Sequencing verified result of species-specific indel visualized by “ggmsa”.

Moreover, we detected the largest species-specific indel, up to 23 bp, situated within the *petL-petG* spacer. To establish reliable barcodes for distinguishing between var. *jujuba* and var. *spinosa*, we designed primers targeting this specific indel. We conducted PCR amplification using collected samples in advance as preliminary validation. Furthermore, we selected six samples (three from each species, labeled as D13 to D15 and S13 to S15) from this set for sequencing to confirm sequence variation. The results, as depicted in Figure 3 and Supplementary Figure S2, revealed clear distinctions between the two species in the sequencing plots generated using “ggmsa” and in the electropherograms, confirming the efficacy of these species-specific identification barcodes.

### 3.3 Phylogeny and divergence time estimation

**FIGURE 4:**
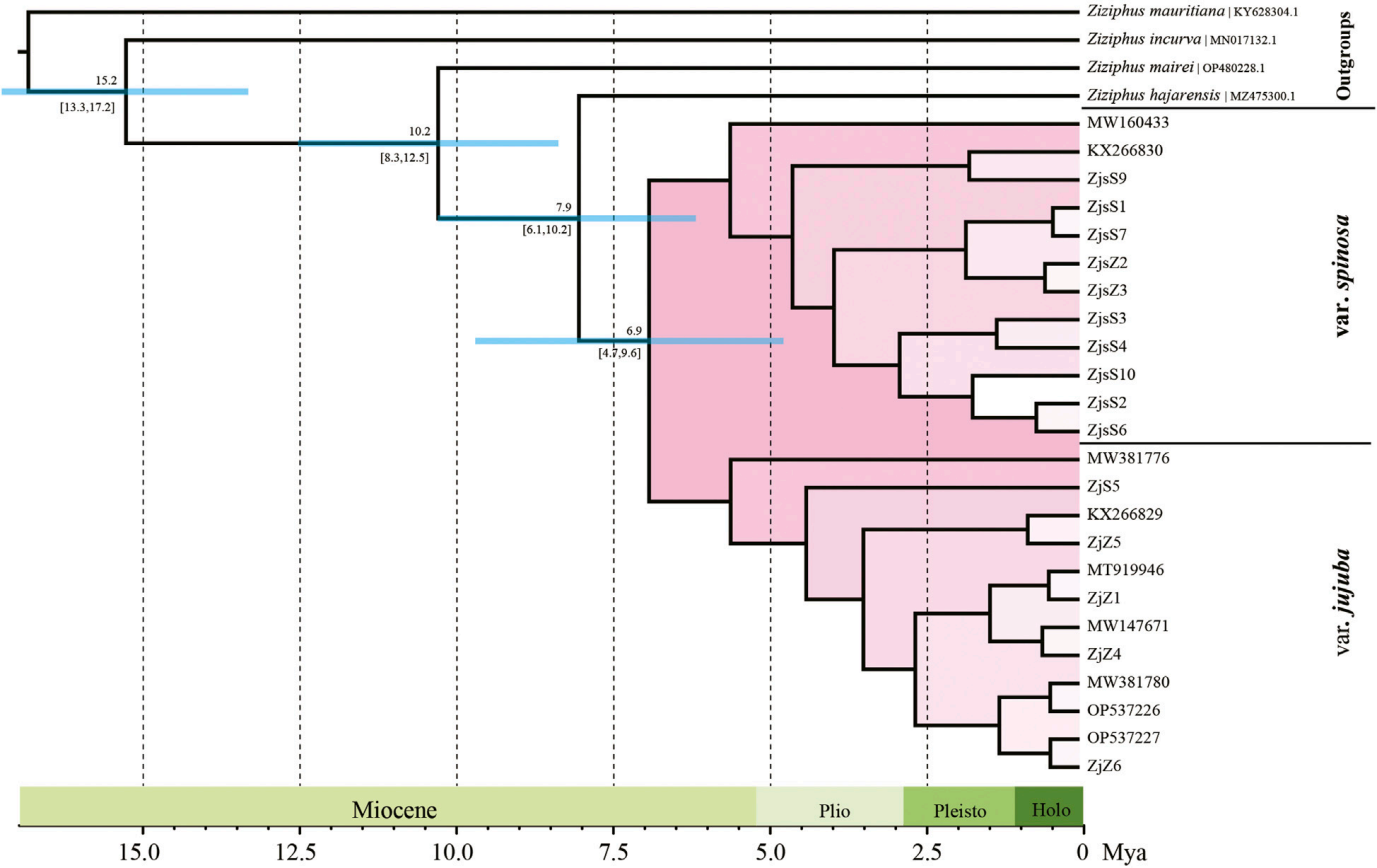
Divergence time of jujube. Numbers above and under the branches indicate the mean divergence times and 95% confidence interval of each node, respectively. Blue bars indicate the 95% highest posterior density intervals.

ML and BI were performed for phylogeny reconstruction based on plastid sequences, taking four *Ziziphus* species as outgroups. The topologies of phylogenetic tree obtained by the two algorithms were almost identical. Accessions of var. *jujuba* and var. *spinosa* formed a monophyletic group with 100% support, and two highly supported clades (BS = 100%, PP = 1.0) within this monophyly corresponding to var. *jujuba* and var. *spinosa* were clearly separated (Supplementary Figure S3). According to the dating result, the divergence of var. *jujuba* and var. *spinosa* from their last common ancestor at around 7.9 Ma (95% HPD: 6.1–10.2 Mya), and later, with a subsequent split from each other in the late Miocene, at 6.9 Ma (95% HPD: 4.7–9.6 Mya) (Figure 4). The diversification time of two species was largely coincidence with the period of dramatic global temperature fluctuations.

### 3.4 Dominant climatic variables and habitat prediction

The MaxEnt model was used to predict the suitable habitats of var. *jujuba* and var. *spinosa*. The average test AUC for the replicate runs was 0.835 with a standard deviation of 0.021 in var. *jujuba* and var. *spinosa* was 0.904 with a standard deviation of 0.014. These high AUC values affirm the efficiency and reliability of the MaxEnt model in forecasting suitable habitats.

**FIGURE 5:**
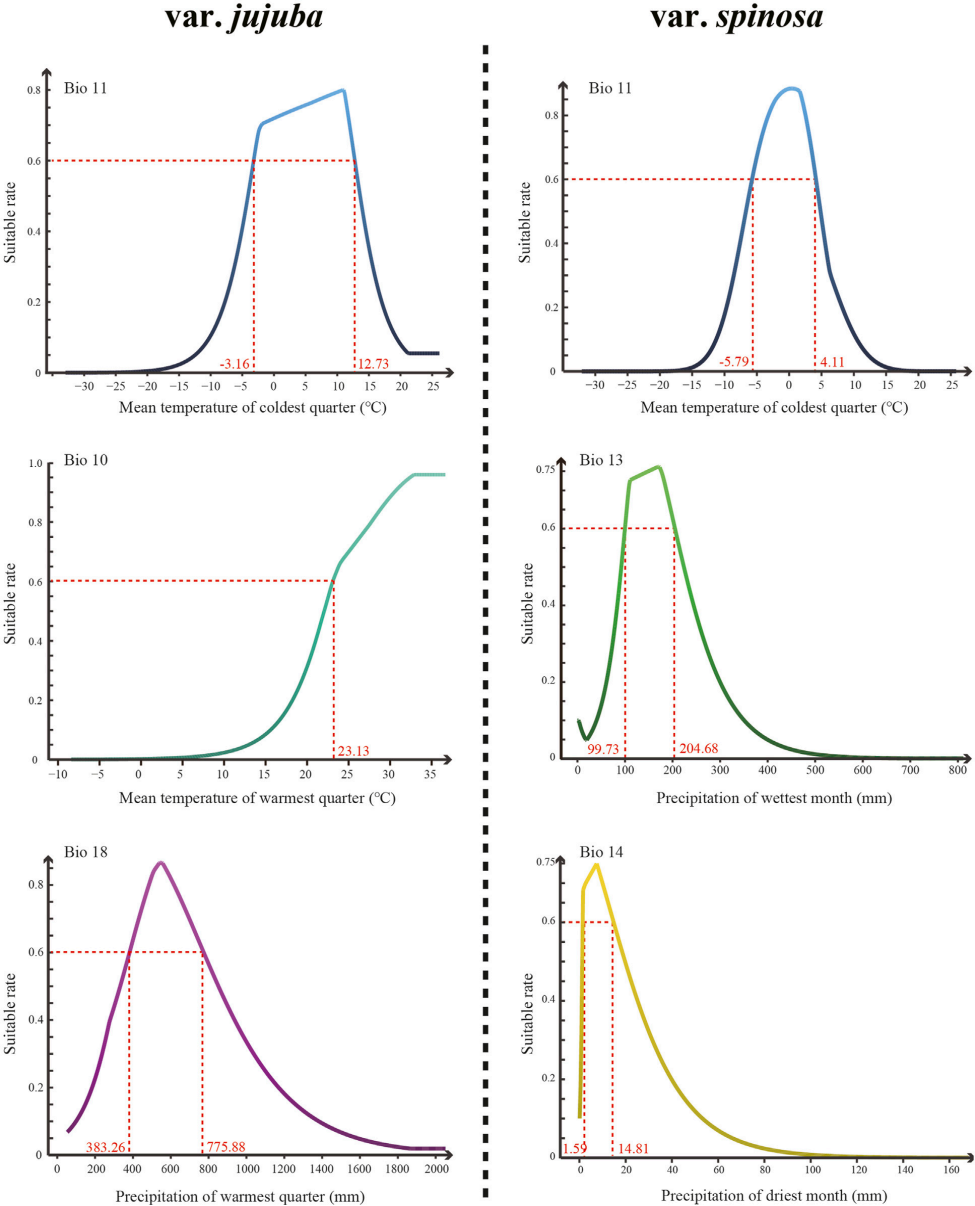
The response curve of the top three most dominant climatic variables of each species generated by the MaxEnt model.

The top three dominant climatic variables of each species were listed in Table 1. For var. *jujuba*, the most influential variable was the mean temperature of the coldest quarter (Bio 11), contributing to 56.5%. It was followed by the mean temperature of the warmest quarter (Bio 10) with a contribution rate of 20.9%, and precipitation of the warmest quarter (Bio 18) at 17.0%. In the case of var. *spinosa*, the top three dominant climatic variables were mean temperature of the coldest quarter (Bio 11, 34.8%), precipitation of the wettest month (Bio 13, 23.2%), and precipitation of the driest month (Bio 14, 16.9%). It is evident that Bio11 significantly influenced the potential suitable habitat of both species. However, there’s a noteworthy distinction: temperature plays a more critical role in shaping the suitable habitat for var. *jujuba*, while var. *spinosa* demonstrates a more balanced response to precipitation and temperature in habitat distribution. The response curve of these dominant climatic variables was shown in Figure 5, illustrating the relationship between species presence probability and climatic variables. To define highly suitable growth conditions, we considered a suitability rate higher than 0.6 as the criterion. Correspondingly, for var. *jujuba*, the optimum ranges of mean temperature of coldest quarter should range from −3.16°C to 12.73°C, mean temperature of warmest quarter should be more than 23.13°C, and precipitation of warmest quarter would better in the range of 383.26–775.88 mm. And for var. *spinosa*, the optimum ranges of mean temperature of coldest quarter should range from −5.79°C to 4.11°C, precipitation of wettest month was more appropriate to be in the range of 99.73–204.68 mm, and precipitation of driest month would better in the range of 1.59–14.81 mm.

**TABLE 1:** Contributions of dominant climatic factors to distribution ranges of the two varieties of *Ziziphus jujuba*.

Taxon	Variable	Climatic factor	Contribution (%)
var*. jujuba*	Bio11	Mean temperature of coldest quarter	56.5
	Bio10	Mean temperature of warmest quarter	20.9
	Bio18	Precipitation of warmest quarter	17.0
var. *spinosa*	Bio11	Mean temperature of coldest quarter	34.8
	Bio13	Precipitation of wettest month	23.2
	Bio14	Precipitation of driest month	16.9

**FIGURE 6:**
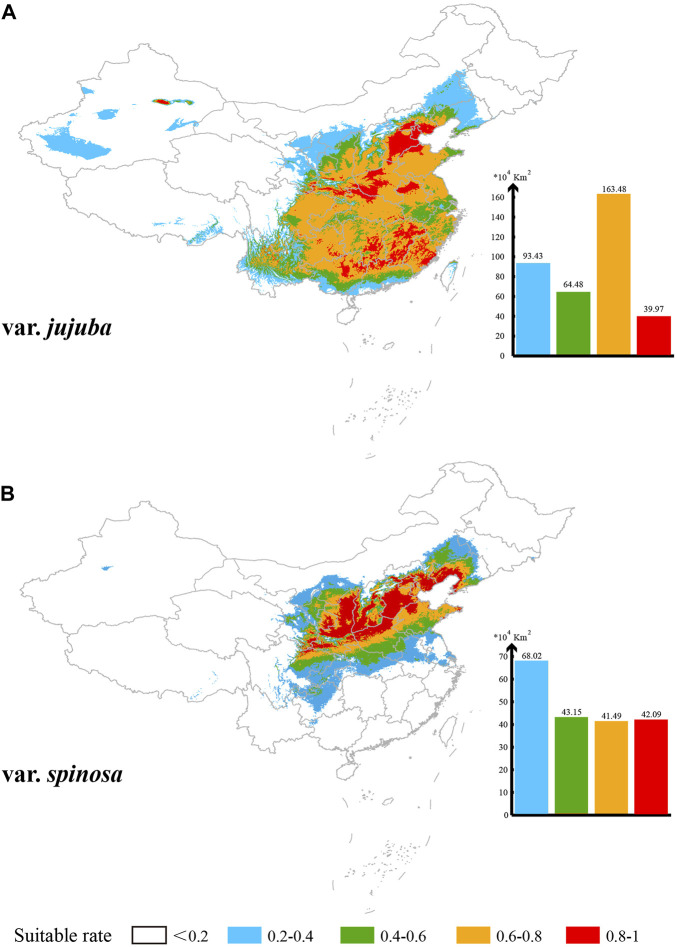
Suitable distribution simulation of these two species in China **(A)** for var. *jujuba* and **(B)** for var. *spinosa*. Five levels of suitability are shown in different colors as follows: no suitability (0–0.2, white); low suitability (0.2–0.4, blue); medium suitability (0.4–0.6, green); high suitability (0.6–0.8, yellow) and ultrahigh suitability (0.8–1, red).

Based on the MaxEnt model’s projections, the potential suitable habitat area in China (suitability rate >0.2) spans 361.36 × 10^4^ km^2^ for var. *jujuba*, primarily distributed south of the Hu Huan-yong Line in China. Meanwhile, the suitable habitat area for var. *spinosa* covers 194.75 × 10^4^ km^2^, predominantly centered in the north-central region of China, with some presence in the southwest and northeast (Figure 6). The areas classified as “ultrahigh suitability” (suitability rate >0.8) are scattered for var. *jujuba*, totaling 39.97 × 10^4^ km^2^ within its suitable habitat. Conversely, var. *spinosa* demonstrates a more concentrated presence, particularly on the Loess Plateau and the North China Plain, covering 42.09 × 10^4^ km^2^ in total. In summary, there exists a notable distinction in the suitable distribution of these two species, with var. *jujuba* having a broader distribution, while var. *spinosa* exhibits a more concentrated habitat.

## 4 Discussion

### 4.1 Genetic diversity and evolutionary history

Genetic diversity serves as the bedrock for the preservation and effective utilization of medicinal plants (Sun et al., 2021). In this study, our comparative analysis of var. *jujuba* and var. *spinosa* reveals a notable absence of significant differences in genome size, gene number and order, GC content, and junction boundaries, implying a remarkable conservation in plastid genome structure. However, their genetic diversity varies. It is well known that domestication is an artificial selection process wherein individual plants with desirable properties are bred to develop varieties that can better meet human needs (Wang et al., 2022c). Generally, wild resources possess higher genetic diversity than domesticated ones, because only a limited number of the best lines are used for breeding the next-generation, which greatly reduces the genetic diversity (Zhou et al., 2015; Liu et al., 2020). But, in terms of the current data, the genetic diversity of var. *jujuba* (so-called domesticated resources) is higher than that of var. *spinosa* (wild), rather than the wild resources having significantly higher genetic diversity.

The results of phylogenetic analysis and dating have elucidated the evolutionary history of these two species. Both ML and BI algorithms strongly support the subclades within these two species. Speciation events are estimated to have occurred during the late Miocene and early Pliocene, approximately 6.9 million years ago. This timing coincides with a significant global temperature fluctuation and extensive glaciation across continents (Zachos et al., 2008). The resultant unique climatic conditions in that period likely facilitated the divergence and speciation of numerous taxa, such as *Pueraria* (Sun et al., 2023), *Leonurus* (Wang et al., 2023), *Arnebia* (Sun et al., 2022), *etc.*, which may also include these two jujube.

Historically, there has been some ambiguity regarding the origins of var. *jujuba*. While some propose that var. *jujuba* was domesticated from var. *spinosa* (Guo et al., 2020), yet others believe that it was domesticated from its wild ancestor, not sour jujube (Shen et al., 2021). Taking into account maternal inheritance, our findings align with the latter perspective, indicating a clear species relationship between these two variants. Nevertheless, resolving this question comprehensively may necessitate more extensive sampling and further exploration.

### 4.2 Rapid species-specific identification

The advent of large-scale, standardized sequencing of the mitochondrial gene CO1 has made people realized that DNA barcoding is an efficient species identification tool (CBOL Plant Working Group, 2009). For plant, plastid gene fragments of *rbcL* and *matK*, as well as *trnH-psbA* and *ycf1* has been widely used. With the burgeoning accumulation of sequence data, DNA barcoding has transcended its role in taxonomy and species identification, branching into diverse fields such as ecology, conservation biology, medicine, and biosecurity (Fišer Pečnikar and Buzan, 2014). However, most current DNA barcodes require sequencing to access individual variation. Even with efficient sequencing services, results take a minimum of 1 day to generate, with time and cost escalating exponentially as sample sizes increase. To expedite species identification, a novel PCR-based Indel marker identification method has gained prominence. This method enables direct identification through the analysis of gel profiles of PCR-amplified fragments and is progressively finding applications in crop and medicinal plant discrimination (Guo et al., 2019; Jain et al., 2019; Kim et al., 2019; Fang et al., 2021).

As it is mentioned, var. *jujuba* and var. *spinosa*’s seedling morphological characteristics is often hindered by habitat conditions, rendering accurate identification challenging. This, in turn, impacts rootstock selection, yield, and medicinal quality. In the current study, we have developed a species-specific barcode marker founded on a 23 bp indel situated in the spacer region of *petL-petG*. Additionally, it is noteworthy that the single-copy feature of the plastid genome lends itself well to the development of species-specific identification markers. Upon initial validation, this barcode has proven adept at effectively discriminating between these two species. Notably, the time required for identification is a mere 3 h, and the cost can be contained to less than two-yuan, significantly savings in time and cost compared to sequencing-based barcodes. Consequently, these innovative PCR-based Indel markers hold substantial promise for rapid species and germplasm classification within var. *jujuba* and var. *spinosa*. This development is poised to make a meaningful contribution to the regulation of the traditional Chinese medicine market. It is important to emphasize that while our results affirm the marker’s efficacy for rapid authentication based on available plastomes data, further evaluation of its accuracy necessitates additional samples from these two species.

### 4.3 Conservation and utilization

The suitable habitat for var. *jujuba* is notably extensive, encompassing subtropical to warm temperate regions, signifying its potential as a lucrative commercial crop in these areas. This underscores the opportunity for rural income generation through its cultivation. Conversely, var. *spinosa* exhibits a more concentrated distribution in North China, particularly in proximity to Chengde, Hebei province. This aligns harmoniously with its recognized “Dao-di” regions in traditional Chinese medicines (TCM). The discernible contrast in suitable habitats emphasizes the need for distinct conservation and utilization strategies for these two species. The influence of dominant climatic variables, as indicated by their contribution weights in our findings, underscores the significance of temperature in shaping the habitat suitability for var. *jujuba*. On the other hand, var. *spinosa* demonstrates a greater tolerance for low precipitation conditions and exhibits superior drought resilience. This resilience, coupled with its well-developed root system, positions it as a pioneer species for desert sand fixation, notably contributing to land reclamation efforts (Shen et al., 2021).

As it is mentioned, these two varieties both effectively serve dual purposes as food and medicine, and have high economic value. However, they are difficult to distinguish, especially during the seedling stage. Mistakes in seedling identification leading to incorrect planting and usage can potentially have adverse effects on the commercial market. Additionally, using sour jujube as rootstock can reduce the incidence of JWB diseases to some extent, and the selection of rootstock also requires accurate identification. Therefore, the development of Indel markers in this study empowers us with a heightened ability to distinguish between var. *jujuba* and var. *spinosa*, and will benefit further utilization. Furthermore, the knowledge of each species’ favorable climate conditions can inform both *ex situ* and *in situ* conservation strategies, aiding in the selection of ideal germplasm nursery sites. Moreover, this information proves invaluable in guiding cultivation and introduction efforts, enhancing the overall management and utilization of these invaluable plant species.

## 5 Conclusion

In this study, we conducted a comparative analysis to unveil the genetic diversity of both var. *jujuba* and var. *spinosa*, in addition to developing novel PCR-based Indel markers for species-specific identification. Furthermore, we embarked on reconstructing the phylogeny and divergence time of *Z. jujuba* and its closely related species, thereby shedding light on their intricate evolutionary history. The divergence of these two species occurred at the late Miocene, potentially influenced by dramatic global temperature oscillations. Additionally, we harnessed MaxEnt modeling to predict the suitable habitats and associated climatic variables for these two species, offering insight into their distinct climate preferences. These revelations foreshadow the necessity of formulating tailored conservation strategies for each species. In brief, our result will improve the further research on these two valuable species and will benefit germplasm resource conservation and utilization.

## Data Availability

The datasets presented in this study can be found in online repositories. The names of the repository/repositories and accession number(s) can be found in the article/Supplementary Material.
