# Supplementary figures and images for: Comparative analysis of jujube and sour jujube gave insight into their difference in genetic diversity and suitable habitat
Source: Front Genet. 2024 Feb 6;15:1322285. doi: 10.3389/fgene.2024.1322285 (PMC10878421; doi:10.3389/fgene.2024.1322285)

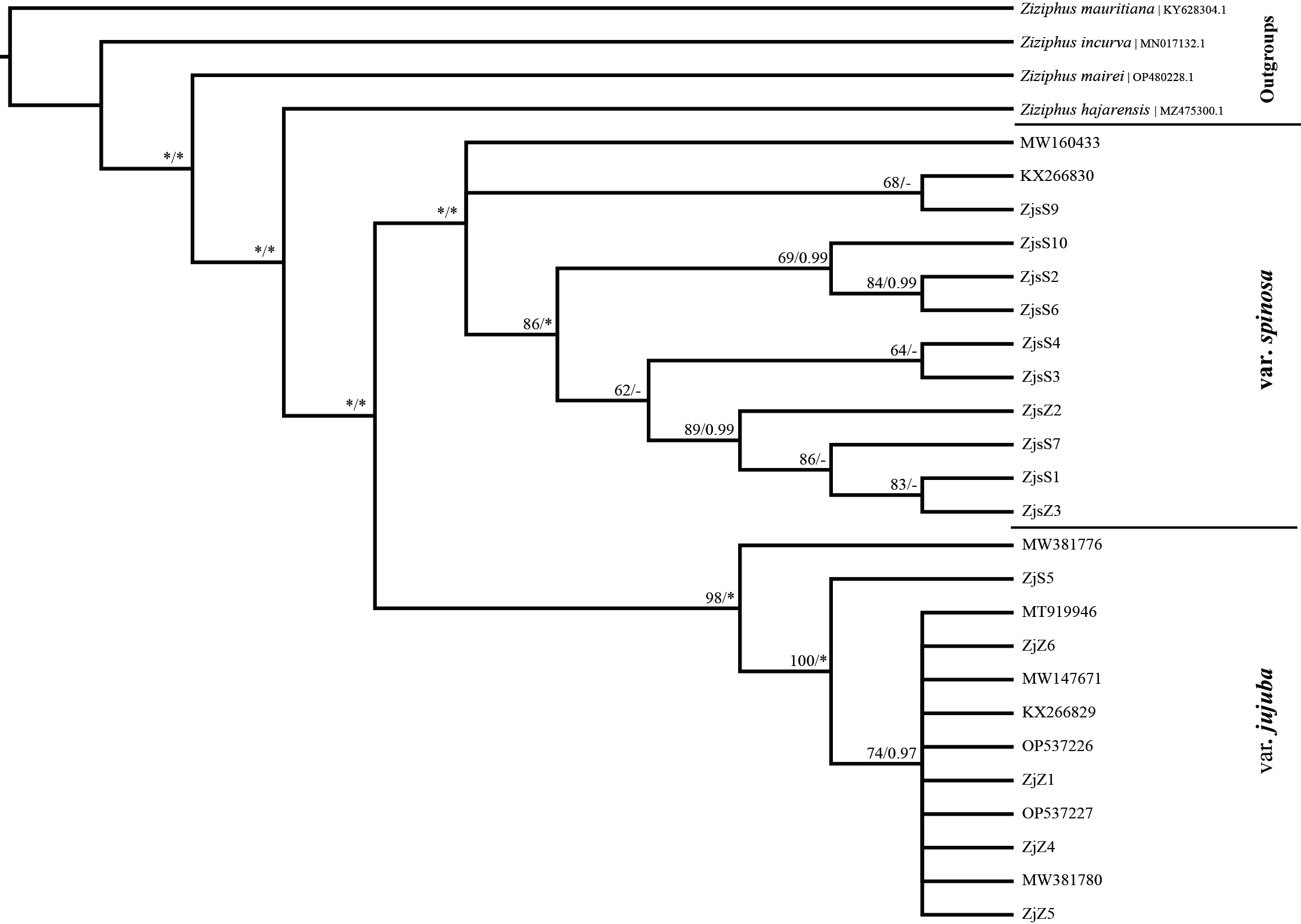

Supplement: Supplementary file 2 [file Image3.jpg]

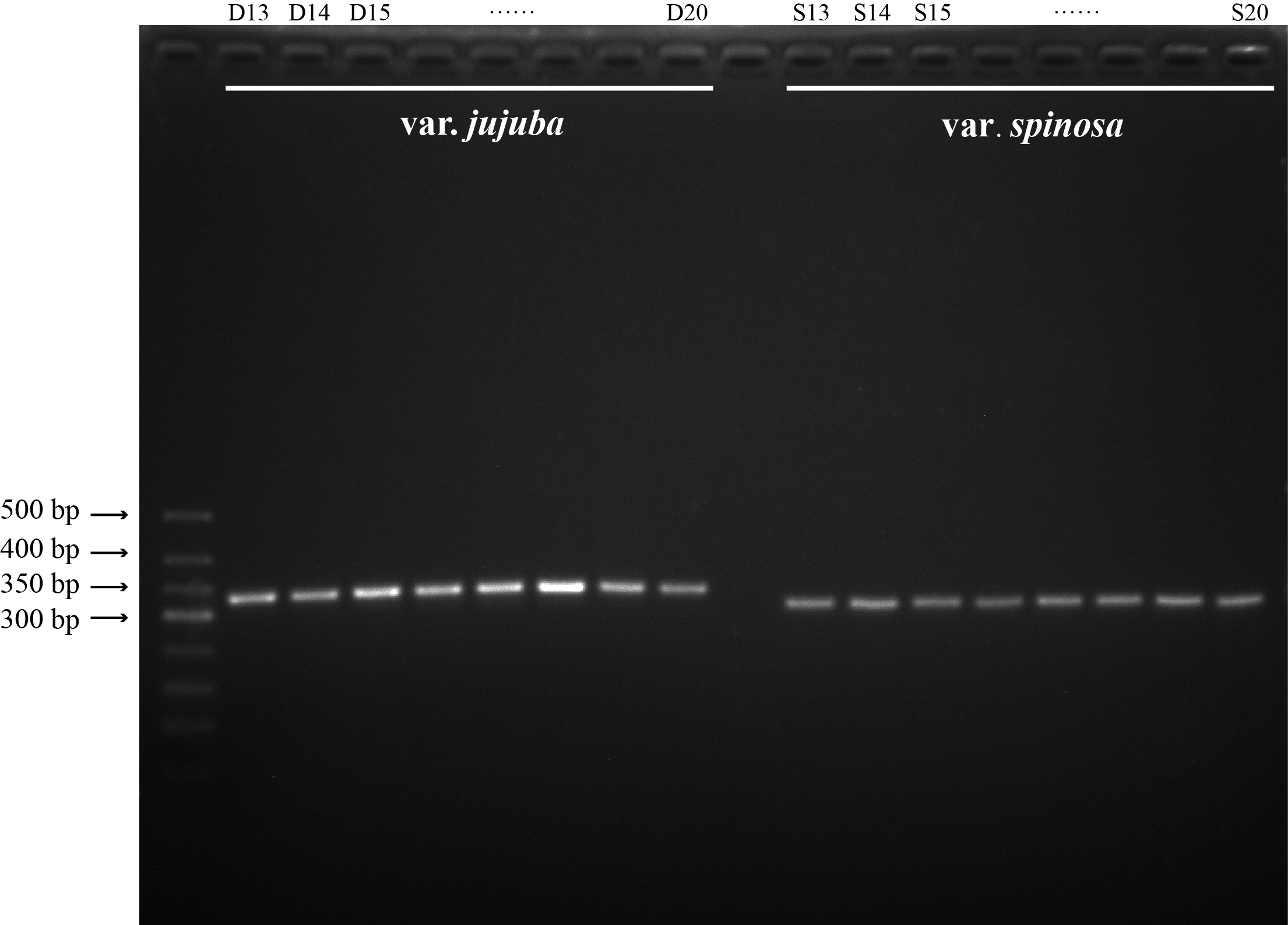

Supplement: Supplementary file 3 [file Image2.jpg]

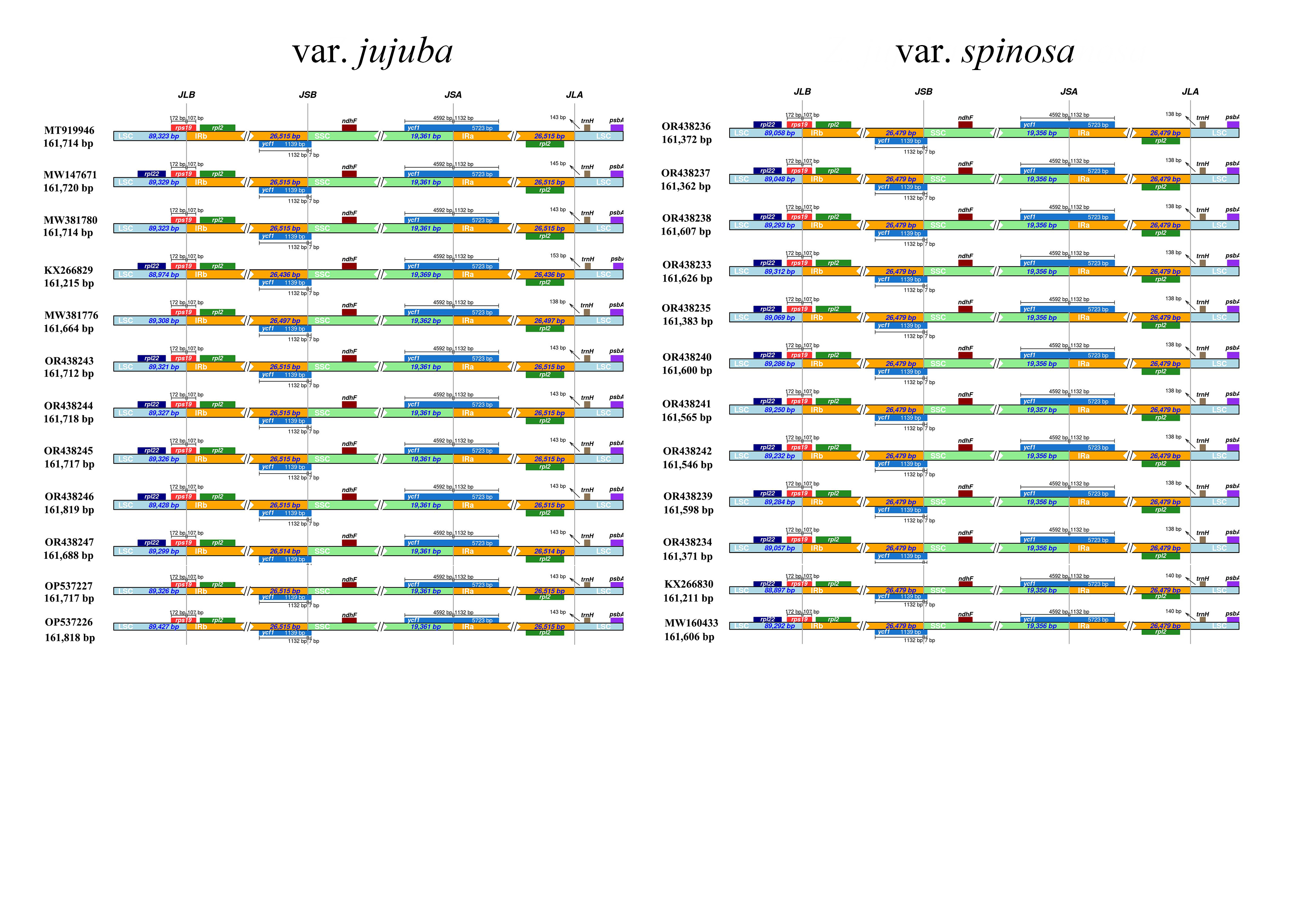

Supplement: Supplementary file 5 [file Image1.jpg]
